# Assessing Drug Product Shelf Life Using the Accelerated Stability Assessment Program: A Case Study of a GLPG4399 Capsule Formulation

**DOI:** 10.3390/pharmaceutics16111400

**Published:** 2024-10-30

**Authors:** Dattatray Modhave, Sara Vrielynck, Kevin Roeleveld

**Affiliations:** 1 CMC Analytical, Galapagos NV, Generaal De Wittelaan L11 A3, 2800 Mechelen, Belgium; 2 Analytical, AnaBioTec NV, Noorwegenstraat 4, 9940 Evergem, Belgium

**Keywords:** stability testing, ASAP, degradation kinetics, shelf life, chemical stability

## Abstract

**Objective:** To evaluate and project the shelf life of GLPG4399, an early-phase clinical drug formulation by applying the Accelerated Stability Assessment Program (ASAP) approach. **Methods:** Forced degradation conditions were implemented to identify the stability-limiting degradation product. The drug and its degradation products were separated using a validated liquid chromatography method. Then, the selected clinical capsule formulation was placed in a glass vial and exposed to accelerated short-term conditions of combinations of high- and low-level heat and humidity in an open state for 5 weeks. The liquid chromatography results were evaluated using the ASAP, which is based on the moisture-modified Arrhenius principle. The resulting data were fitted using a suitable diffusion kinetics method. **Results:** The developed model was applied to predict the shelf life of the drug product when using clinically appropriate primary packaging (high-density polyethylene container). The derived stability parameters of the moisture-modified Arrhenius equation were the Arrhenius collision frequency, activation energy, and humidity sensitivity constant. The goodness of fit parameters R^2^ (>0.95) and goodness of prediction Q^2^ (>0.80) parameters for the selected model were acceptable. The results of the accelerated, short-term stability study were verified against real-time, long-term 12-month data. **Conclusions:** We demonstrated the application of the ASAP approach to evaluate the shelf life of a GLPG4399 solid capsule formulation. The studied ASAP approach can be extended to evaluate the stability and shelf-life estimations of other early-phase clinical formulations.

## 1. Introduction

Stability testing is conducted to ensure the quality of a pharmaceutical product during its storage and to assign an appropriate shelf life. Understanding physical and chemical stability principles is essential in driving product safety and efficacy [1]. Chemical degradation of drugs in a solid state often follows nonlinear and complex kinetics. Specifically, oxidation-mediated degradation is a highly complicated reaction [2,3]. Reactive impurities (residual peroxides, reactive oxygen species, and inorganic metals) are difficult to avoid in the excipients, and even a trace amount of these impurities can adversely alter product quality during storage [4].

During stability studies, specific environmental conditions are applied for a predetermined period of time. Conventional stability studies are carried out as stated in the International Council for Harmonisation quality guidelines (ICH Q1A [R2]) [5]. In the product development cycle, protocol-driven, long-term stability testing requires a significant amount of time and resources, and involves substantial costs. The health authorities encourage the exploration and implementation of alternative approaches to support stability studies. Over the past few years, many such science- and risk-based approaches have been developed, particularly for solid drug products [6,7,8,9,10,11,12,13,14]. In the last decade, the International Consortium for Innovation and Quality in Pharmaceutical Development (IQ) formed a working group to focus on and build a roadmap for the use of Risk-Based Predictive Stability (RBPS) tools. The working group discussed several case studies that are used during the regulatory submission process. Considering the acceptance from many regulatory authorities, it is clear that future stability designs will utilize predictive stability approaches to support the traditional ICH stability program [12,13,14]. Waterman provided an overview of the application of the Accelerated Stability Assessment Program (ASAP) for predicting the shelf life of a solid oral drug formulation [15]. Since then, the ASAP*prime* software, version 6.0, has been widely implemented in the pharmaceutical industry to support accelerated stability testing of compounds, particularly for small molecules. Based on the understanding that it takes longer for molecules to degrade under mild conditions, the approach of the ASAP is to mimic the chemical drug degradation process using accelerated conditions.

The ASAP uses the isoconversion concept, which is defined as the time to reach the specification limit for the stability-limiting degradation product at a given temperature and humidity (i.e., the time to fail). An experimental ASAP design includes exposure of the drug product to various combinations of temperatures and humidities to accelerate degradation over a shorter period of time (3–5 weeks). The appropriate statistical approach and best-fit kinetics model are utilized to propose a shelf life of the drug product. In addition, the ASAP approach can be used to provide a rationale for selecting the appropriate primary packaging configuration. It is less time-consuming and resource-intensive than the conventional ICH accelerated stability approach and can be implemented in the clinical phase settings to determine the shelf life of a product and assess various primary packaging configurations. For solid drug products, several studies using the ASAP*prime* software were explored by Malcolm [16]. Primarily, these studies demonstrated the successful application of the ASAP for justifying stability-induced out-of-specification results of tablet drug formulations [16].

In general, computing simulation methodologies offer a promising way of supporting seamless manufacturing and stability testing of drug products by speeding up the drug development process and providing cost-effective solutions for pharmaceutical development. The regulatory agencies have recommended modernized drug development approaches implementing in silico modeling and statistical tools in (pre)clinical, manufacturing, and stability testing stages. Pharmacokinetic/pharmacodynamic modeling is reported as one of the successful examples [17,18]. From a regulatory standpoint, besides ICH, other regions have streamlined the stability guidelines and associated principles. The lean stability designs are perfectly aligned with the quality by design (QbD) approach. Such approaches help to gain product understanding, speed up the decision process, and build the right control strategies during drug development. Accelerated stability studies can be used as a risk assessment tool during the post-approval changes while filing minor or major variations [19,20,21]. During the early phase clinical setting, due to the shorter development timeline, it is important to guarantee the shelf life of the developed formulation for the desired primary packaging. The main objectives of the studies were identifying the stability-limiting degradation product of the test formulation, implementing a short-term ASAP design to predict the shelf life of a selected packaging configuration and, lastly, verifying the predicted outcomes against the real-time long-term storage conditions. These objectives were outlined and accomplished for the GLPG4399 clinical capsule formulation.

## 2. Materials and Methods

Chemical oxidative reagents (hydrogen peroxide [H_2_O_2_] solution [30% *w*/*w*] and 2,2′-azobis[2-amidinopropane] dihydrochloride [AAPH]) were purchased from Sigma-Aldrich (St. Louis, MO, USA). Ultrapure water was obtained from a TKA (Waltham, MA, USA) water purification unit. Acetonitrile and methanol were of high-performance liquid chromatography (HPLC) grade, and all other chemicals and salts used were of analytical reagent grade. The prototype GLPG4399 formulation used was a hydroxypropyl methyl cellulose-based capsule filled with a powder blend composed of pharmaceutical-grade excipients and an active test compound (active drug). The formulation uses the dry granulation unit processing operation. The drug content of the selected GLPG4399 drug product capsule was 25 mg, with a drug loading of 5% *w*/*w* (95% excipient loading). The capsules were packaged in 50 mL high-density polyethylene (HDPE) bottles with polypropylene caps. The desiccant bags contained silica gel (Brownell, London, UK). Standard glass vials were used during the accelerated, short-term storage exposure, and HDPE bottles were used for the real-time, long-term storage study.

### 2.1. Forced Oxidative Degradation/Stress Study in the Solution State

Due to the oxidative labile functional group (aryl ether type) present in the chemical structure of GLPG4399, the forced degradation study focused on oxidative stress-mediated reactions. The stress study was carried out separately using 5 mM AAPH (free radical initiator) reagents to induce oxidative stress in the active pharmaceutical ingredient (API) at 40 °C and liquid H_2_O_2_ (0.3% *v*/*v*) to induce oxidative stress in the API at ambient temperature. A mixture of methanol and water (90:10) was used as a diluent for preparing the oxidative stressors. The active test compound was dissolved in each of the oxidative reagent solutions and exposed for a period of 2 days at ambient temperature for H_2_O_2_ and at 40 °C for AAPH (free radical initiator). After exposure, samples were withdrawn and analyzed using liquid chromatography (LC). This preliminary forced degradation study was performed to identify the stability-limiting degradation product.

### 2.2. Accelerated, Short-Term Storage Stability Study

The accelerating factors for drug degradation are temperature and humidity. The experimental design involved exposing the drug product samples to variable temperatures (50–70 °C) and extreme humidities (10–80% relative humidity [*RH*]) for a short duration of time, typically for up to 5 weeks and at different time intervals.

As a standard protocol, the drug product capsules were placed in glass vials. To achieve the desired humidity, an appropriate saturated salt solution (sodium chloride, sodium bromide, calcium sulfate, potassium carbonate, or potassium chloride) was placed in the bottom of a desiccator and covered by a perforated plate [22]. The glass vials containing the drug product were placed in the desiccator and were exposed in the open state (without a lid). The desiccator was then transferred to a thermal oven set at a set, controlled temperature. The five specific temperature/humidity combinations tested were 50 °C/50% *RH* (2 and 5 weeks); 50 °C/75% *RH* (1, 2, and 5 weeks); 60 °C/10% *RH* (2 and 5 weeks); 60 °C/80% *RH* (1, 2, and 5 weeks); and 70 °C/40% *RH* (2 and 5 weeks). An additional condition of 40 °C/75% *RH* was applied for 2 weeks. Humidity values were recorded and plotted using a calibrated data logger (Labguard 3D, bioMérieux, Lys, France). As part of the excipient compatibility screening protocol, a feasibility study was also carried out to select the storage conditions for further investigation. For the storage conditions investigated, the intention was to accelerate the chemical degradation process without altering the physical state of the product (solid polymorphic form). To perform this, glass vials containing the powdered blend formulation (without a capsule shell) were exposed to selective stress conditions of 40 °C/75% *RH* and 60 °C/75% *RH* for 2 weeks (open vials without a lid). The feasibility study outcomes helped to select the suitable ASAP conditions.

The stored samples were pulled from chambers and analyzed using the LC method, and the degradation data were further statistically evaluated using the ASAP*prime* software.

### 2.3. Real-Time, Long-Term Storage Stability Study

As outlined in the ICH guideline, the health authorities request confirmation of the predicted or proposed shelf life to support the recommended storage conditions and packaging. In the present study, the applied storage conditions were ambient temperature and humidity (15–25 °C and 40–55% *RH* [<60% *RH*]), and the packaging material was HDPE bottles. Fifteen drug product capsules were added to HDPE bottles (50 mL), which were then closed with the caps and placed in stability cabinets. The real-time, long-term stability study was carried out in temperature and humidity chambers set at 25 ± 3 °C and 60 ± 5% *RH* (KBF 720, BINDER, Tuttlingen, Germany). The bottles were stored in the chambers and were withdrawn and analyzed at regular intervals of 1, 3, 6, and 12 months. The LC method described in Section 2.4 was applied to analyze the samples and report the data.

### 2.4. Establishment of the LC Method and Analysis of Samples

LC analyses were performed using a Waters (Milford, MA, USA) HPLC system equipped with a photodiode array detector. Empower 3.0 software was used for data processing and acquisition. The oxidative stress samples from the solution-state, forced-degradation study (using peroxide and free radical media) were used to develop the HPLC method. This method comprises a mobile phase system containing a mixture of aqueous (10 mM ammonium acetate [pH = 4.0], labeled as mobile phase A) and organic (acetonitrile, labeled as mobile phase B) phases. The ratios of mobile phase solvent were varied to achieve sufficient resolution between the drug and degradation product. A gradient elution program was set at a flow rate of 0.5 mL/min at different time intervals (T_min_/mobile phase A%): T_0_/95; T_1_/95; T_16_/5; T_18_/5; T_18.01_/95; and T_24_/95. The injection volume was set at 1 μL. The reversed-phase LC method was used with a C18 BEH phenyl column with a 1.7 µm particle size, 2.1 mm internal diameter, and 100 mm length. The limit of quantification and limit of detection of the method were 0.05% and 0.02%, respectively. The area under the curve (of a chromatographic peak) for the degradation product was used to assess the extent of degradation in terms of the area percentage. The LC method was selective and linear over the concentration range of 0.25 µg/mL–0.6 mg/mL. For the developed method, repeatability was confirmed in replicate samples (*n* = 3). The LC method validation parameters are summarized in Table S1.

A mixture of acetonitrile and water in a ratio of 50:50 *v*/*v* was selected as a diluent system for sample preparation. The capsules were placed in a volumetric flask, and the required amount of diluent was added to dissolve the test compound and achieve a final concentration of 0.5 mg/mL.

### 2.5. Statistical Modeling and Data Analysis

Many statistical programs have been evaluated to model the degradation kinetics and predict the shelf life of pharmaceutical products; among these, a commonly used approach is the ASAP. Primarily, ASAP uses the concept of isoconversion (the time to cross the specification limits for a degradation product at a given temperature and humidity) and is based on the moisture-modified Arrhenius principle (Equation (1)):

(1) $$ln\;k=lnA-\frac{Ea}{RT}+B\left(RH\right)$$

where *k* is the degradation rate (percentage of degradation product generated per day), *A* is the Arrhenius collision frequency (pre-exponential factor), *B* is the humidity sensitivity constant, *Ea* is the activation energy of the degradation reaction, *T* is the temperature in Kelvin, *R* is the gas constant (1.986 cal/[mol K]), and *RH* is the percentage of relative humidity. Arrhenius kinetics refers to the linear dependence of the natural logarithm of the reaction rate *k* on the *RH* and the reciprocal of the absolute temperature *T*. The ASAP methodology uses a regression approach to determine the ln *A*, *Ea*, and *B* terms to estimate the drug product shelf life as a function of the recommended storage temperature and humidity.

For GLPG4399, the obtained degradation data for each of the tested accelerated, short-term conditions were fitted by applying the suitable kinetic model to extrapolate the shelf life under the real-time 25 °C/60% *RH* condition and with HDPE bottles as the primary packaging configuration. During the data evaluation, the supplier-specified (Gerresheimer, Düsseldorf, Germany) moisture vapor transmission rate (0.27 mg/day/bottle at 25 °C/60% *RH*) was used for the selected HDPE bottle as an input parameter. In addition, for the packaging material that was evaluated, the shelf life was estimated and compared based on the presence (2 g) and absence (0 g) of desiccant in the bottle configuration. The commercially available ASAP*prime* version 6.0 software was used. This software performs Monte Carlo simulations to estimate confidence intervals for the predicted shelf life. Lastly, the predicted shelf life was verified and reported against the real-time, long-term stability data generated for the test drug product capsule formulation.

**Figure 1:**
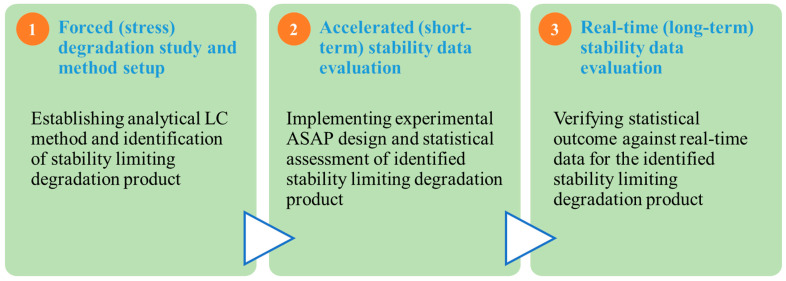
Summary of experimental setup and workflow.

The typical workflow followed during this experimental study and statistical evaluation is summarized in Figure 1.

## 3. Results and Discussion

### 3.1. Forced Degradation Outcomes

The primary objective of the forced degradation study was to identify the stability-limiting degradation product. For the AAPH-injected sample, the distinct peak observed in the chromatographic run at the relative retention time of 1.10 provided evidence of drug degradation. The observed degradation levels were 0.61% and 1.20% on days 1 and 2, respectively. The data reflect the susceptibility and time-dependent oxidation under this condition. The chemical structure of the degradation product was identified using LC-mass spectrometry (LC-MS). The obtained mass data and structure elucidation confirmed the oxidative degradation reactions (the chemical structures of the drug and its oxidative degradation product, and the associated structural elucidation are not disclosed in this manuscript). The mass method parameters are described in the Supplementary Material (Method S1). The oxidative degradation product at the relative retention time of 1.10 was labeled as DP-O. The absence of any additional chromatographic peak in the control sample (in the absence of an oxidative reagent) provided further evidence that the drug degradation in the AAPH-injected sample occurred as a function of the stressor. In addition, the absence of a chromatographic peak at the relative retention time of 1.10 in the H_2_O_2_-stressed sample demonstrated the selectivity of the drug toward free radical-assisted oxidative degradation over direct H_2_O_2_-induced oxidation. Furthermore, the visual appearance was darker for the AAPH-treated samples than for the control samples (untreated drug solution).

### 3.2. Accelerated, Short-Term and Real-Time, Long-Term Stability Outcomes

**Figure 2:**
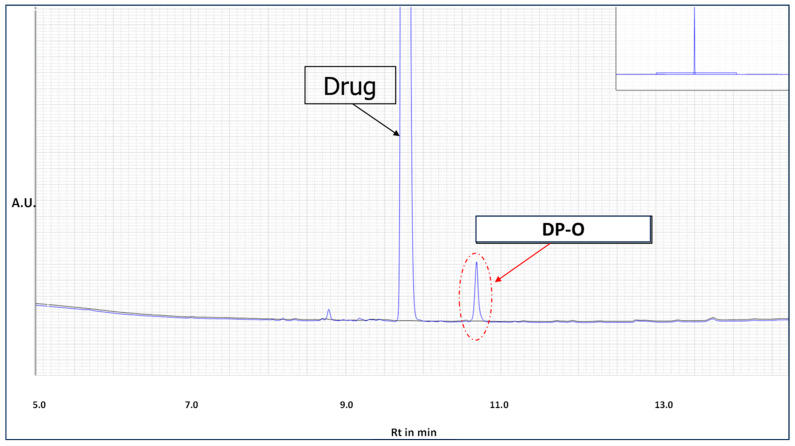
Representative chromatogram highlighting the retention times of the drug (active moiety) and the stability-limiting degradation product (DP-O). The black line represents the diluent system solvent.

The samples exposed to the accelerated and real-time storage conditions were withdrawn and analyzed using the LC method. The percentage degradation observed in each of the conditions is captured in Table 1. The chromatographic peaks for the drug and the DP-O are shown in Figure 2.

During the feasibility evaluation, in the two examined temperature-humidity conditions, DP-O was formed. A temperature-dependent increase was observed for the DP-O stability-limiting degradant. At higher temperature conditions (40 °C/75% *RH*), the level of degradation product was 0.04% which went to 0.08% at 60 °C/75% *RH*.

As shown in Table 1, DP-O formed in each of the accelerated storage conditions; however, the extent of degradation was greater in dry conditions than in humid conditions. In addition, the percentage DP-O level increased with the exposure time. The observations revealed that the compound has a higher susceptibility to oxidative stress in dry conditions than in humid conditions. The trace-level free radical reactive excipient impurities were attributed as a potential cause of drug oxidation in the solid capsule formulation. Case studies have reported that the common excipient contains free radical impurities and have described the detrimental drug degradation effects associated with these impurities [23,24]. The polymeric excipients in the selected capsule formulation likely carry a risk of trace-level reactive oxidative impurities.

In the subsequent step, the obtained chemical degradation data were used for statistical evaluation (deriving Arrhenius parameters) and estimating the shelf life using the ASAP*prime* software.

The real-time, long-term data indicate that the drug was stable until 6 months when the percentage DP-O level observed was less than the limit of detection (0.02%). At the 12-month time point, a small amount of DP-O (0.07%) was noted. The obtained real-time data were fitted using the developed model.

As additional information, during the short-term stability study, three representative two-week stored test samples (40 °C/75% *RH*, 60 °C/80% *RH*, and 70 °C/40% *RH*) were assessed using powder X-ray diffractometry. The obtained diffractogram was found to be consistent with the initial results (at T0, these are samples that were not exposed to temperature and humidity), indicating no change in the physical polymorphic state (form data not disclosed in this manuscript). Water activity was also measured during long-term storage conditions. The initial water activity was recorded as 0.4, which increased to 0.5 by the end of the storage condition (12 months, 40 °C/75% *RH*). This indicates the low hygroscopic nature of the studied formulation.

### 3.3. Statistical Evaluation

In ASAP*prime*, the accelerated, short-term data were used to derive the degradation rate based on the obtained Arrhenius parameters (e.g., *Ea* and *B*) and to estimate the shelf life for the capsule formulation. The percentage of stability-limiting degradation product level (% DP-O) was modeled and used to estimate the product shelf life. The acceptable specification limit of 0.20% recommended by the ICH was applied for the DP-O. During storage of the samples, a period of up to 5 weeks was set to achieve sufficient degradation, preferably crossing the set specification limit. The estimated ln *A*, *Ea*, and *B* parameters are shown in Table 2. The obtained *B* value suggests lower sensitivity of the drug product in humid than dry conditions. The high *Ea* indicates greater stability (less temperature sensitivity) of the compound (Figure S1) [25].

**Figure 3:**
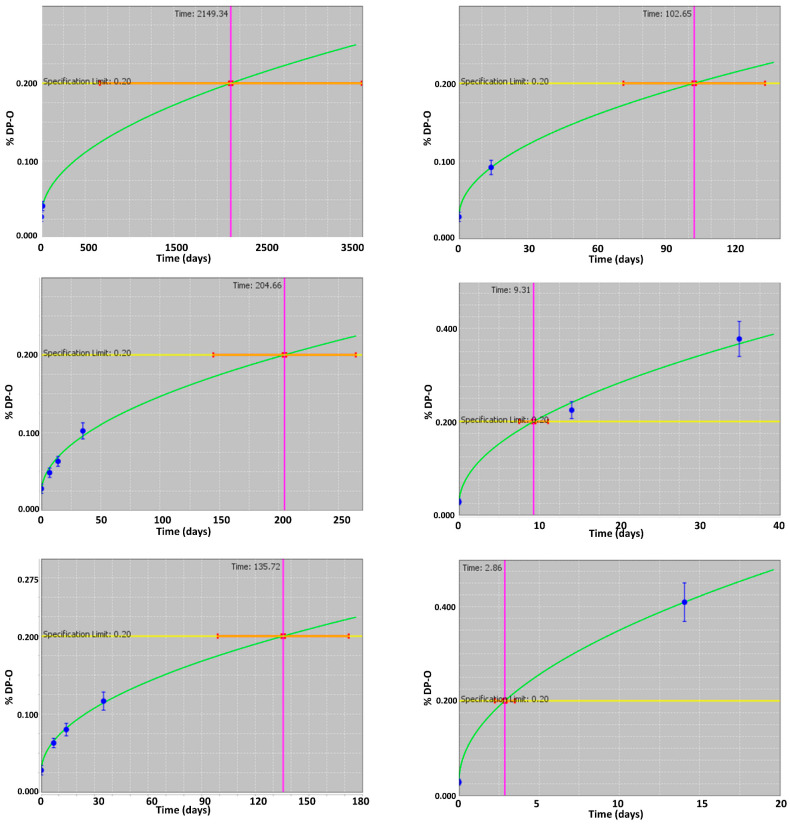
Individual isoconversion plots showing the percentage degradation product (DP-O) level over time (in days) under the studied conditions: 40 °C/75% RH (**top-left**); 50 °C/50% *RH* (**top-right**); 50 °C/77% *RH* (**middle-left**); 60 °C/10% *RH* (**middle-right**); 60 °C/80% *RH* (**bottom-left**); and 70 °C/40% *RH* (**bottom-right**). The yellow line indicates the targeted specification limit. The green line represents the model best fit through the available data points (blue dots) for %DP-O. The pink line indicates the time to reach the isoconversion time. Orange lines are the error bars for the isoconversion time. Blue dots and lines plot the % DP-O level with error bars.

The isoconversion paradigm was applied in the ASAP*prime* software to predict the shelf life of the drug product. For each assessed storage condition, the individual isoconversion graphs (percentage DP-O level versus time in days) are presented in Figure 3. Initially, linear zero and first-order kinetics were applied to the data; however, R^2^ and Q^2^ values were observed to be <0.90 and <0.70, respectively. Both models were subsequently eliminated due to poor representation of drug degradation (plateau formation) and their limited predictive power. Thus, the software’s in-built diffusion kinetics method was applied. Diffusion kinetics behaviors are expected for the oxidative degradation mechanism, in which the rate-determining step is diffusion-dependent, especially for solid drug products [3,24,26,27]. The obtained goodness of fit (R^2^ > 0.95) and goodness of prediction (Q^2^ > 0.80) parameters indicated the acceptable precision of the chosen diffusion kinetics [28]. The model was also subsequently evaluated and verified using the long-term data. Altogether, the results demonstrated that the diffusion kinetics model was the robust choice for accurate shelf-life prediction. The mathematical form of the diffusion equation is detailed in the Supplementary Material (Equation (S1)).

In the early clinical development phase, HDPE bottles are considered an easy and convenient primary packaging configuration for supplying drug products at the clinical study center. In the present study, two packaging conditions were considered and assessed for shelf-life prediction during statistical evaluation: HDPE bottles with and without desiccant. For the predicted shelf life, the obtained probabilities of passing were 99.60% (for 3 years) and 95.20% (1.5 years) with and without desiccant, respectively. A probability of passing of more than 95% is generally considered acceptable. As seen in the LC results, the percentage DP-O level was lower in dry conditions than in humid conditions; this observation aligned with the predictions for the packaging without desiccant. The observation can be linked to the oxidative degradation mechanism, where the dried state would have favored the higher chemical conversion. The other plausible reason is the greater availability of oxidative free radical species in the dry state compared with the moist state [24,26,29]. The primary packaging with desiccant provides a dry environment and is expected to protect drug products that are sensitive to external moisture. However, in the present study, the use of desiccant was not favorable. For the studied capsule drug product, an initial shelf life of 12 months was aimed for and was considered sufficient based on the planned clinical study duration and the predicted outcome using HDPE bottles without desiccant.

For GLPG4399, the final selected packaging material for the storage and supply of the capsule formulation was an HDPE bottle without desiccant. For this primary packaging, it was expected that the percentage DP-O level would remain within the specification limit (<0.20%) throughout the expiry period in the recommended storage conditions (<25 °C/60% *RH*). The real-time, long-term stability data were subsequently generated using this packaging condition at 25 °C/60% *RH*.

**Figure 4:**
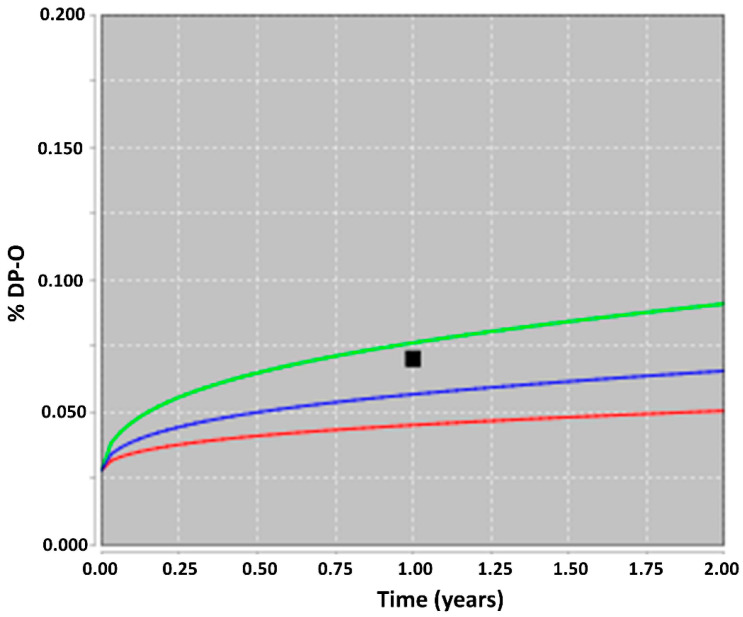
Real-time percentage DP-O levels at 25 °C/60% *RH* over time (in years) compared with the model predicted plot. Blue line: average predictions; green and red lines: ± standard deviation. The black square denotes the long-term, real-time data (1-year data).

In the second step, the obtained real-time, long-term stability data for the DP-O were used to verify the predicted data. As shown in Table 1, in HDPE bottles (without desiccant), the percentage DP-O level remained within the set specification limit for up to 1 year of storage. At 12 months of storage, the percentage DP-O level was above the limit of quantification but well below the target specification limit (0.20%), and the drug was therefore within its estimated shelf life. Figure 4 illustrates the long-term, real-time data fitted against the modeled predicted data. The results demonstrated that the real-time data were consistent with the predictions made using ASAP*prime*, showing excellent agreement. The long-term, real-time storage data also validate the predictive power of the implemented model. Table S2 and Figure S2 illustrate the accelerated ICH long-term storage condition (six months at 40 °C/75% *RH*) fitted against the predicted data from ASAPprime. For the long-term, real-time condition (25 °C/60% *RH*), degradation had not yet started, or occurred very slowly, within the first six months and had initiated within the subsequent six months. Multiple time points within six to twelve months would have helped to better understand the trend of degradation. On the other hand, in the accelerated ICH long-term condition, degradation initiated in the first three months and quickened after that. This reflects the temperature/humidity-time dependent relationship for the drug oxidation process. Furthermore, we recognize that the nondisclosed content (API and DP-O chemical structure) would have simplified the understanding of this observed degradation trend.

### 3.4. The Relevance and Practical Application of the Approach

Chemical stability is mainly governed by drug degradation. For solid products in particular, drug degradation is a complex process and seldom follows standard zero (linear) or first-order (exponential) kinetics. Hence, during statistical evaluation, selecting the precise modeling approach and the right kinetic parameters is of the utmost importance in the prediction of the reasonable shelf life. The degradation rate is compound-specific and depends on the *Ea* and *B*. Of the many potential fit methods (e.g., zero, first, and second order, diffusion, and Avrami–Erofeev kinetics), the most appropriate needed to be determined during the statistical evaluation. During the accelerated, short-term stability study, the accuracy of predictions improved with the increasing number of data points selected.

The studied approach can be used to screen prototype formulations and select the right excipient and packaging for the product. From the regulatory perspective, the ASAP can be used as a supportive tool to set the initial shelf life of the clinical formulation and to facilitate post-approval changes of registered products (e.g., minor formulation compositions, excipient grades, and supplier grades). For a given formulation, once the model is established, it can be used to assess the impact of packaging changes. Similarly, the effect of temperature variations that occur during the storage and shipment processes can be assessed using the same principles. Furthermore, the ASAP outcomes can be useful in refining the long-term ICH stability protocols and in reducing the risk of stability-derived product failures. However, as we have demonstrated in the present study, verification using real-time data is necessary to validate the predicted outcome of the accelerated study.

## 4. Conclusions

In the present study, we demonstrated the application of the ASAP approach to evaluating the shelf life of a solid capsule formulation. The assessed solution-state, forced degradation conditions confirmed the susceptibility of the compound to oxidative stress. The applied ASAP Arrhenius kinetics parameters were successfully obtained for the studied formulation. The results of the accelerated, short-term stability study were verified against real-time, long-term data. It was found that drug oxidation progresses faster in dry conditions than in humid conditions. In addition, the results of the packaging assessments revealed that the drug product had a longer shelf life in HDPE bottles in the absence of desiccant than when desiccant was present. Although the number of time points collected from the real-time long-term study were limited, the shelf-life and stability aligned well with the calculations made by the twelve-month model. The studied ASAP approach can be extended to evaluate the stability and shelf-life estimations of other early-phase clinical formulations. It is less time- and resource-consuming and offers an alternative solution to traditional stability studies used in industrial settings. Such science- and risk-based predictive methods will help answer multiple questions that arise while selecting stable formulation, screening protective primary packaging, and setting product expiry.

## Figures and Tables

**Table 1:** Percentage DP-O level during stability exposure.

Stability Condition	Time Duration	Percentage DP-O Level ^a^
Accelerated, short-term stability data		
NA	0 weeks	ND
50 °C/50% *RH*	1 week	NT
	2 weeks	0.0915 ± 0.0005
	5 weeks	0.1628 ± 0.0022
50 °C/75% *RH*	1 week	0.0483 ± 0.0010
	2 weeks	0.0629 ± 0.0020
	5 weeks	0.1022 ± 0.0020
60 °C/10% *RH*	1 week	NT
	2 weeks	0.2245 ± 0.0159
	5 weeks	0.3776 ± 0.0006
60 °C/80% *RH*	1 week	0.0627 ± 0.0027
	2 weeks	0.0800 ± 0.0021
	5 weeks	0.1165 ± 0.0062
70 °C/40% *RH*	1 week	NT
	2 weeks	0.4099 ± 0.0012
	5 weeks	0.8574 ± 0.0037
40 °C/75% *RH*	1 week	NT
	2 weeks	0.0417 ± 0.0014
	5 weeks	NT
Real-time, long-term stability data ^b^		
25 °C/60% *RH*	0 months	ND
	1 month	ND
	3 months	ND
	6 months	ND
	12 months	0.07

^a^ Given as mean ± SD for the accelerated, short-term stability data; given at the RRT of 1.10 for the real-time, long-term stability data. ^b^ The real-time, long-term data are limited to two decimal digits (as provided by the stability source report). DP-O, oxidative degradation product; NA, not applicable; NT, not tested; ND, not detected (below the lower limit of quantification); RH, relative humidity; RRT, relative retention time; SD, standard deviation.

**Table 2:** Arrhenius parameters derived using ASAP*prime* software.

Parameter	Value ± SD (If Applicable)
ln *A*	50.7 ± 11.1
*Ea* Kcal/mol	35.8 ± 7.4
*B*	−0.0330 ± 0.0104
R^2^	>0.95
Q^2^	>0.80

## Data Availability

Data obtained from preclinical Galapagos-sponsored research are unavailable to protect intellectual property rights.

## Supplementary Materials

The following supporting information can be downloaded at: https://www.mdpi.com/article/10.3390/pharmaceutics16111400/s1, Figure S1. Visual representation of *Ea* and *B* using the ASAP*prime*. Figure S2. Percentage DP-O level at 40 ºC/75% *RH* over time compared with the modeled projected fitted plot. Table S1. Liquid chromatography method validation parameters and summary. Table S2. Percentage DP-O levels during long-accelerated stability exposure.
