# Supplementary material for: Assessing Drug Product Shelf Life Using the Accelerated Stability Assessment Program: A Case Study of a GLPG4399 Capsule Formulation
Source: Pharmaceutics. 2024 Oct 30;16(11):1400. doi: 10.3390/pharmaceutics16111400 (PMC11597223; doi:10.3390/pharmaceutics16111400)
Supplement: Supplementary file 1 [file pharmaceutics-16-01400-s001.zip › pharmaceutics-3184653-supplementary.pdf]

Supplementary Materials

*Representation of Activation Energy and Humidity Sensitivity Constant Using ASAP*prime

The critical stability parameters such as activation energy (*Ea*) and humidity sensitivity constant (*B*) are visually represented in Figure S1. The plot shows high *Ea* and *B* values, suggesting reasonable stability of the compound under hot and humid conditions.

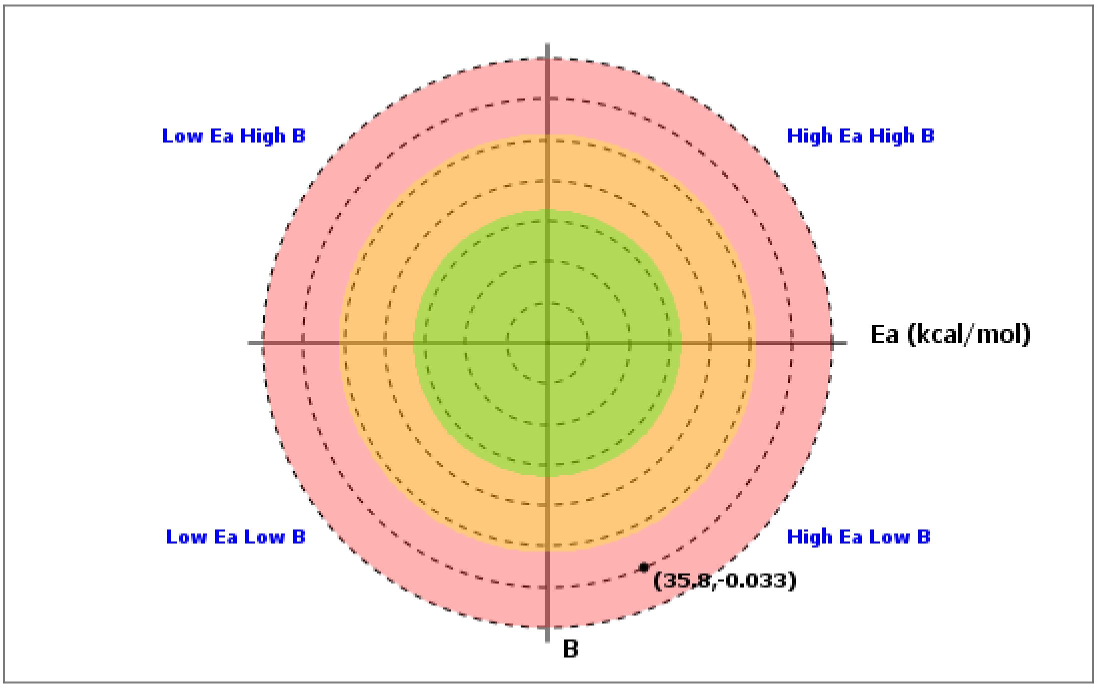

Figure S1. Visual representation of *Ea* and *B* using the ASAP*prime*.

Diffusion Kinetics Description

The mathematical form of the diffusion equation is (Equation (S1)):

$$[deg] = (kt)^{1/2}$$

where *k* is the degradation rate constant and *t* is time. The isoconversion time was defined as the time when the percentage degradation product level equaled or crossed the specification limit. The degradation product data presented in Table 1 (accelerated, short-term conditions) were used for plotting the individual isoconversion plots (temperature and relative humidity), while fitting, some of the extreme points were not included.

To improve the predictive accuracy of the selected fitting method and maximize the goodness of fit (R^2), data from 5 weeks at 70 °C/40% relative humidity (*RH*) and 50 °C/50% *RH* were not included in subsequent analyses.

Liquid Chromatography Method Validation Parameters and Summary

The tested liquid chromatography method validation parameters and acceptance criteria are listed in Table S1.

Table S1. Liquid chromatography method validation parameters and summary.

**Parameter**	**Acceptance Limit**
Specificity	No peaks ≥0.50% are present at the retention time of the drug substance or its degradation products. Alternatively, if present, evaluate the significance of the interference and, if needed, justify blank correction<br>No interference between drug substance and degradation products<br>Excipients are separated from the main compound and from each other
Range and Linearity	For assays at 80–120% concentration levels and with *n* = 3:<br>• correlation coefficient ≥0.998<br>• slope of the calibration curve 0.95–1.05
	For degradation products at 0.05–2.0% concentration levels and with *n* = 3:<br>• correlation coefficient ≥0.99<br>• confidence interval around the intercept includes 0 or ≤5% of area compound of interest<br>• slope of the calibration curve 0.90–1.10
Accuracy	For assays at 80–120% concentration levels and with *n* = 3:<br>• individual recovery 95–105%
	For degradation products at 0.05–2.0% concentration levels:<br>• individual recovery 50–150% (0.05% level)<br>• individual recovery 70–130% (0.5% level)<br>• individual recovery 90–110% (2.0% level)
Precision as Repeatability	For assays at 80–120% concentration levels and with *n* = 3:<br>• average RSD ≤2%
	For degradation products at 0.05–2.0% concentration levels:<br>• average RSD ≤25% (0.05% level)<br>• average RSD ≤15% (0.5% level)<br>• average RSD ≤5% (2.0% level)
	For system precision (*n* = 5):<br>• RSD ≤2%
LOQ	For assays at 0.05% concentration levels and with *n* = 5:<br>• LOQ ≤ reporting threshold (0.05%)<br>• S/N ratio obtained at LOQ ≥10<br>• RSD ≤ 15%
LOD	For assays at 0.02% concentration levels and with *n* = 3:<br>• LOD ≤ reporting threshold (0.05%)<br>• S/N ratio obtained at LOD ≥3

LOD, limit of detection; LOQ, limit of quantification; RSD, relative standard deviation; S/N, signal-to-noise.

Method S1

The LC-MS analysis was conducted using a high-resolution mass spectrometer (Orbitrap Exploris 240, Thermo Scientific, Waltham, MA, USA). A full scan of all masses between 100–1000 m/z with a resolution of 120,000 was performed using electrospray ionization in positive mode.

Statistical Evaluation at long-term accelerated condition

Besides the real-time long-term condition, the study was extended to another accelerated storage condition of 40 ± 3 ºC/75 ± 5% *RH* for six months. The storage intervals were 1, 3, and 6 months. For this extra condition, the oxidative degradation product (DP-O) data is presented in Table S2 and plotted in Figure S2.

Table S2. Percentage DP-O levels during long-accelerated stability exposure.

Stability Condition	Time Duration	Percentage DP-O Level
	Accelerated, ICH long-term stability data	
40 ºC/75% *RH*	0 months	ND
	1 month	ND
	3 months	0.06
	6 months	0.14

ICH, international council of harmonization; ND, not detected; RH, relative humidity.

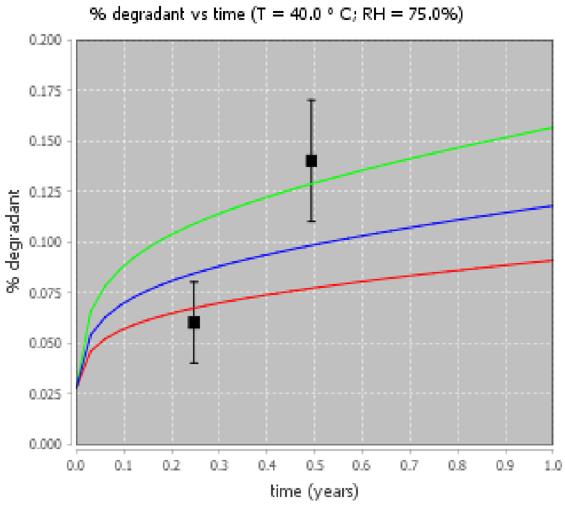

Figure S2. Percentage DP-O level at 40 ºC/75% *RH* over time compared with the modeled projected fitted plot. Blue line: average predictions; green and red lines: ± standard

deviation. The black square denotes the data at 3 and 6 months (derived from the above table).

As shown in Table S2, the degradation initiated in the first three months and sped up after that. This reflects the temperature-/humidity–time-dependent relationship for the drug oxidation process.
